# The Post-Ovariectomy Interval Affects the Antidepressant-Like Action of Citalopram Combined with Ethynyl-Estradiol in the Forced Swim Test in Middle Aged Rats

**DOI:** 10.3390/ph9020021

**Published:** 2016-05-03

**Authors:** Nelly M. Vega Rivera, Alfredo Gallardo Tenorio, Alonso Fernández-Guasti, Erika Estrada Camarena

**Affiliations:** 1 Neuropsicofarmacología, Dirección de Neurociencias, Instituto Nacional de Psiquiatría “Ramón de la Fuente Muñiz”, INPRFM, Calzada México-Xochimilco 101, Col San Lorenzo Huipulco 14370, Mexico; vegquim2909@hotmail.com; 2 Departamento de Farmacobiología, Centro de Investigación y Estudios Avanzados-Sede Sur. IPN, Calzada de los Tenorios 235, Col Granjas Coapa 14330, Mexico; alfie_891@hotmail.com (A.G.T.); jfernand@cinvestav.mx (A.F.-G.)

**Keywords:** ethynyl-estradiol, citalopram, forced swim test, post-OVX interval, perimenopause, antidepressant-like effect

## Abstract

The use of a combined therapy with low doses of estrogens plus antidepressants to treat depression associated to perimenopause could be advantageous. However the use of these combinations is controversial due to several factors, including the time of intervention in relation to menopause onset. This paper analyzes whether time post-OVX influences the antidepressant-like action of a combination of ethynyl-estradiol (EE_2_) and citalopram (CIT) in the forced swim test (FST). Middle-aged (15 months old) female Wistar rats were ovariectomized and after one or three weeks treated with EE_2_ (1.25, 2.5 or 5.0 µg/rat, s.c.; −48 h) or CIT (1.25, 2.5, 5.0 or 10 mg/kg, i.p./3 injections in 24 h) and tested in the FST. In a second experiment, after one or three weeks of OVX, rats received a combination of an ineffective dose of EE_2_ (1.25 µg/rat, s.c., −48 h) plus CIT (2.5 mg/kg, i.p./3 injections in 24 h) and subjected to the FST. Finally, the uteri were removed and weighted to obtain an index of the peripheral effects of EE_2_ administration. EE_2_ (2.5 or 5.0 µg/rat) reduced immobility after one but not three weeks of OVX. In contrast, no CIT dose reduced immobility at one or three weeks after OVX. When EE_2_ (1.25 µg/rat) was combined with CIT (2.5 mg/kg) an antidepressant-like effect was observed at one but not three weeks post-OVX. The weight of the uteri augmented when EE_2_ was administrated three weeks after OVX. The data suggest that the time post-OVX is a crucial factor that contributes to observe the antidepressant-like effect of EE_2_ alone or in combination with CIT.

## 1. Introduction

Women report more depression than men (by a factor of 2:1), particularly associated with their reproductive life span [1]. In fact, the onset or exacerbation of depressive symptoms has been associated to perimenopause [2,3]. Some reports indicate that the antidepressant response could be modified by the endocrine condition, since premenopausic women are more responsive than post-menopausic females [4,5,6]. However, other reports do not show differences in the response to antidepressants during the perimenopause transition [7,8]. Important methodological differences could contribute to explain these controversial results; for example, the time of intervention in relation to menopause onset, called the “critical period” [9,10] as well as whether menopause is natural or induced. In this line, recent reports suggest that an opportune intervention during early menopause is more effective than during late-menopause [11,12,13,14,15]. However, Henderson and Popat [16] in a systematic review, reported that the literature is not enough to support or decline this hypothesis.

Estrogen replacement therapy (ERT) is an effective treatment for depression associated to perimenopause, along with its beneficial effects on other climacteric symptoms like osteoporosis and hot flashes [17]. The use of a combined therapy of antidepressants plus ERT to treat depression and climacteric symptoms seems useful. For example, ERT may shorten the onset of the therapeutic effects of antidepressants [5,18,19,20] and may reduce vasomotor symptoms, prevent osteoporosis and cardiovascular insults, as well as promoting neuroprotection [21,22,23]. However, controversial results have restricted their utilization [17,24], among them the efficacy of ERT to reduce depression [8,25] *versus* their putative adverse effects. For example, the combined use of ERT plus antidepressants has been related to a higher risk of developing cancer, particularly in long-term users and in women with a history of breast cancer [26,27]. In contrast, short-term treatment with a low-dose of conjugated equine estrogens and fluvoxamine is effective and safe for oophorectomized women in relieving hot flashes and depression [28]. Preclinical data in models that have predictive value, using young rats and at a constant post-OVX interval, showed that estrogens shorten and potentiate the antidepressant-like action of selective serotonin reuptake inhibitors (SSRIs) [21,29]. However there are no studies exploring whether age and the post-OVX interval modify the antidepressant-like action of the combination of estrogens and antidepressants.

Interestingly, the antidepressant-like effects of estrogens appear to depend on both the age and post-OVX interval [30,31,32,33]. For instance, 5 or 10 μg/rat of 17 β-estradiol (E_2_) produced an antidepressant-like action in 3 month old OVX rats, while in 15 month old rats, only 10 μg/rat had an effect; suggesting that E_2_ sensitivity decreases with age [34]. Furthermore, no action was reported in senescent rats [30,31]. Interestingly, aging also abbreviates the intervention window with E_2_, since the point of restitution in relation to time after OVX is three months after OVX in young rats, while in aged rats it is restricted to one week [13,32,33,34]. The action of antidepressants is also modulated by age, because their effect decreases in middle-age rats and may vanish at senescence [34,35,36].

Therefore, the aim of the present study was to explore whether the post-OVX interval influences the antidepressant-like action of 17α-ethynyl estradiol (EE_2_) and citalopram (CIT) alone or in combination in the forced swim test in rats aged 15 months. As an index of EE_2_’s activity in peripheral tissues, the uterus weight was evaluated [32,37]. Citalopram was chosen because of its wide use in clinical practice due to less pharmacokinetic interactions with other drugs [38,39], particularly in middle-aged women that frequently consume several drugs [23]. EE_2_ was selected because it is an estrogen commonly used in ERT, is more potent than E_2_ in reducing immobility behavior in the FST [40] and possesses a wider window of effect in the FST after OVX [32].

## 2. Results

### 2.1. Experiment 1: Effect of Post-OVX Interval on the Effect of EE_2_ or CIT in Middle-Aged Rats on the FST

**Figure 1:**
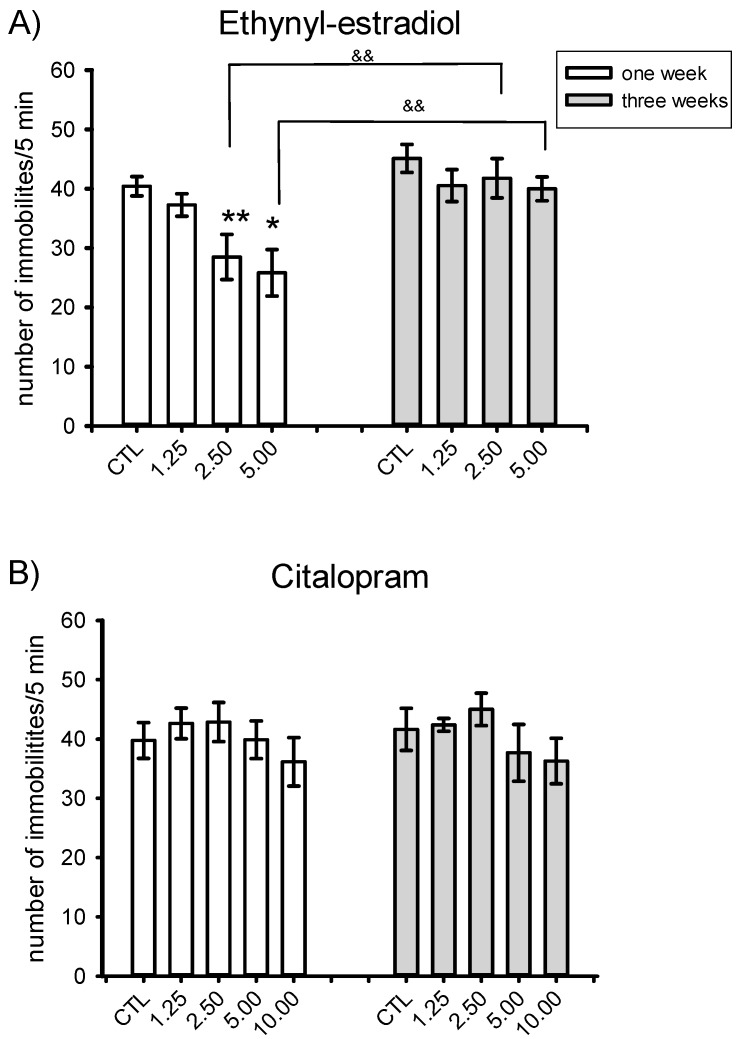
(**A**) Effect of ethynyl estradiol (1.25, 2.5 or 5 µg/rat; −48 h before the test; *n* = 7–9 per group); and (**B**) citalopram (1.25, 2.5; 5.0 or 10 mg/kg; −23, −5, −1 h before the test; *n* = 6–8 per group) at one or three weeks after ovariectomy in the forced swim test. The data represent the mean ± S.E.M. of the number of immobilities scored in intervals of 5-s during a 5-min test session. Holm-Sidack test * *p* < 0.05; ** *p* < 0.005 *versus* respective control group; ^&&^ *p* < 0.005 *versus* three weeks.

Figure 1 shows the effect of several EE_2_ doses (panel A) administered 1 or 3 weeks post-surgery on behavioral immobility in the FST. The two-way analysis of variance indicated differences for the factor time [F(1,51) = 23.81, *p* < 0.001] and dose of EE_2_ [F(3,51) = 4.23, *p* = 0.009] without a significant interaction between them [F(3,51) = 1.25, ns]. *Post-hoc* comparisons revealed that at 1 week post-OVX (Panel A), EE_2_ reduced the immobility behavior compared to the control group at doses of 2.5 µg/kg (*p* = 0.008) or 5.0 µg/kg (*p* = 0.04). However, at 3 weeks after OVX, no dose of EE_2_ induced changes on immobility. Differences between one and three weeks were observed at the doses of 2.5 or 5.0 µg/kg (*p* = 0.002 and *p* = 0.001, respectively).

Figure 1B shows that no CIT dose tested reduced immobility at one or three weeks after OVX. The two-way analysis of variance showed no significant effect of time [F(1,63) = 0.02, ns]; dose of CIT [F(4,63) = 1.64, ns] or the interaction between these factors [F(4,63) = 0.14, ns].

Table 1 shows the effect of EE_2_ or CIT on the active behaviors scored in the FST. EE_2_, at 5.0 µg/rat, increased swimming only one week after OVX (*p* < 0.001). This increase differed from the value of the control group and the group treated with EE_2_ at this dose but tested three weeks post-OVX (*p* < 0.001). The two way ANOVA values for swimming behavior were: time post-OVX [F(1,54) = 21.76, *p* < 0.001], treatment [F(3,54) = 21.76, *p* = 0.02] and the interaction [F(3,54) = 4.18, *p* = 0.01]. No changes were observed in climbing behavior in response to EE_2_ treatment at any time post-OVX. Thus the two way ANOVA values for climbing behavior were: time post-OVX [F(1,54) = 1.78, ns], treatment [F(3,54) = 0.98, ns] and their interaction [F(3,54) = 2.45, ns]. On the other hand, no CIT dose changed swimming or climbing behavior at any time post-OVX.

Table 2 shows the weight of uteri in 15 month old rats treated with different doses of EE_2_ (1.25, 2.5 or 5.0 µg/rat, s.c. −48 before FST) at one or three weeks post-OVX. The uteri from rats treated acutely with oil three weeks after surgery were lighter than those from rats treated with oil after one week (*p* = 0.03). The groups that received EE_2_ at one week post-OVX did not show significant changes in their uteri weight. In contrast, the groups that received a single dose of EE_2_ at 2.5 or 5.0 µg/rat had significantly increased uteri weight compared to the control group (*p* < 0.05) and to the groups that received the same dose one week after OVX (*p* < 0.001). The two-way ANOVA revealed no significant differences for the factors time [F(1,23) = 0.19, ns] or dose [F(3,23) = 0.65, ns]; however, a significant interaction between them was detected [F(3,23) = 7.66, *p* < 0.001].

### 2.2. Experiment 2: Effect of Post-OVX Interval on the Effect of the Combination of Non-Effective Doses of EE_2_ plus CIT in Middle-Aged Rats on the FST

**Figure 2:**
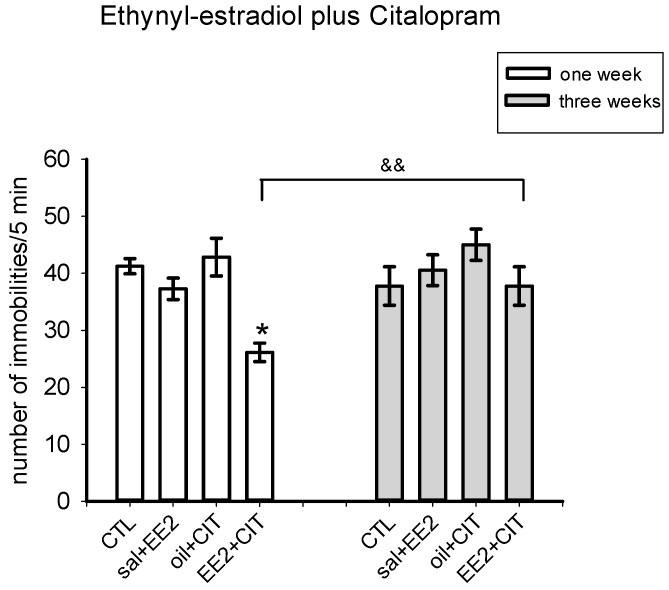
Effect of the combination of ethynyl estradiol (2.5 µg/rat; −48 before the test) plus citalopram (2.5 mg/kg; −23, −5, −1 h before the test) at one (*n* = 6–8 per group) or three (*n* = 6–8 per group) weeks after ovariectomy in the forced swim test. The data represent the mean ± S.E.M of the number of scored immobilities in intervals of 5-s during a 5-min test session. Holm-Sidack test * *p* < 0.05versus respective control group; ^&&^ *p* < 0.005 *versus* three weeks.

Figure 2 shows the effect of a combined low dose of EE_2_ (1.25 µg/kg) and CIT (2.5 mg/kg) administered at one or three weeks post-OVX on the FST. EE_2_ or CIT alone did not modify the immobility behavior in the FST at one or three weeks post-OVX. In contrast, the combination EE_2_ plus CIT tested one week after OVX decreased immobility compared to the control group (*p* < 0.05) and *versus* the same treatment at three weeks post-OVX (*p* < 0.001), when it failed to have an effect. The two-way ANOVA showed significant differences for time [F(1,51) = 5.25, *p* = 0.02]; no significant disparity for treatment [F(3,51) = 3.35, *p* = 0.08] and a significant interaction between these factors [F(3,51) = 2.77, *p* = 0.05].

Table 3 shows the effect of the combination of low doses of EE_2_ plus CIT on swimming and climbing scored in the FST. EE_2_ in combination with CIT increased swimming at one, but not at three, weeks post-OVX compared to the control group and the group treated three weeks after OVX. The two way ANOVA yielded the following values for time post-OVX [F(3,51) = 2.47, *p* = 0.07]; treatment [F(1,51) = 2.48, *p* = 0.09] and their interaction [F(3,51) = 5.50, *p* = 0.002]. No significant changes were observed on climbing behavior in response to EE_2_ plus CIT.

## 3. Discussion

The antidepressant-like action of EE_2_, in contrast to that of CIT, depends on the post-OVX interval. Remarkably, the combination of a non-effective dose of EE_2_ plus CIT promotes an antidepressant-like action in the FST only one week post-OVX. In middle-aged female rats, the antidepressant-like effect of EE_2_ was observed at one but not at three weeks post-OVX. These results are partially in line with previous data showing that ERT produces an antidepressant-like effect in FST if given closer to the estrogen decline [30,32]. However, the present findings are in contrast with a previous report where EE_2_ exerted an antidepressant-like effect in the FST after twelve weeks of OVX [32]. An explanation for this divergence is the age of the females: young (3 months) *versus* middle-aged (15 months) rats. In support, some reports indicate that ovariectomized young (3 months) and adult (7 months) rats are more responsive to E_2_ restitution than middle-aged animals (12 months) [30,31]. At the age of twelve months none of the female rats shows regular estrous cycles most have irregular cycles and a few show persistent estrus or diestrus [41,42,43]. After reproductive senescence, there could be an adjustment in estrogen receptor sensitivity [44,45,46] that affects the critical period for an intervention with estrogens. Importantly, most studies perform OVX before periestropause and evaluate the critical window for intervention considering the period of estrogen decline [12,33,45,47], but not the age at which OVX is performed. In this sense, recently García *et al.* [47] showed that changes in the expression of genes involved in social and affiliative behaviors—such as vasopressin and oxitocin—varied according to the age when OVX was performed as well as the interval after OVX.

CIT lacked an effect on the FST independently of the post-OVX interval. Several reports indicate that CIT produces an antidepressant-like effect in the FST [48,49]; however these studies were performed in 3 month old rats. It could be argued that the doses we tested were insufficient to promote an antidepressant-like effect. However, Flores Serrano *et al.* [48] showed that CIT reduced immobility on the FST at doses of 1 or 3 mg/kg, in 2–3 month old female rats independently of the estrous cycle. In the present study, doses in the range of 1.25 to 10 mg/kg had no action, making it unlikely that a dose-related problem explains the absence of an effect. Most likely, age and endocrine condition are factors that contribute to decrease the sensitivity to CIT. A similar result was reported by Olivares-Nazario *et al*. [35] who showed that senescent (23–25 months) male rats did not respond to the antidepressant-like effect of desimipramine or fluoxetine in the FST. In contrast, middle-aged females (14 to 18 month old), subjected to the chronic mild stress depression model, showed a transitory antidepressant-like effect of 10 mg/kg CIT [50]. Differences in the model and treatment schedule (chronic *versus* sub-acute) could contribute to the divergent results. However, the transitory effect of CIT in the chronic mild stress paradigm [50] suggests some degree of age-dependent insensitivity.

CIT is a highly selective serotonin transporter (SERT) inhibitor [49]. A previous study showed that SERT expression increased in response to stress by FST in young but not in middle-aged rats [51]. Indeed, studies in non-human primates have shown that aging is associated with decreased specific SERT binding [52]. Accordingly, it is possible that CIT lacked an antidepressant-like effect due to reduced SERT sensitivity caused by aging [53] and the endocrine condition. Supporting this notion, the present study showed that the combination of a non-effective dose of EE_2_ plus a low dose of CIT produced an antidepressant-like action after one week of OVX, suggesting that EE_2_ modulates SERT activity. In agreement, earlier reports indicated that the activity and affinity of SERT are modulated by estrogens, such as EE_2_ [54,55,56], even in males. Interestingly, the antidepressant-like action of EE_2_ requires presynaptic elements, possibly the SERT [57].

Alternatively, the combination of EE_2_ plus CIT maybe is effective one week after OVX because EE_2_ acts at estrogen receptors, which indirectly modulate the serotonergic system through 5-HT1A receptors [57,58,59]. The present data agree with a previous report showing that the combination of EE_2_ plus a SSRI mainly affected swimming behavior [49], which is regulated by the serotonergic system [60].

Importantly, the modulation that EE_2_ exerts on the effect of CIT has a critical period after which it is ineffective. The factors that underlie this response are unknown; however, it has been reported that estradiol requires the ERα-mGlutR1, PI3K/Akt and MAPK/ERK1/2 pathways to modulate the activity of SSRIs on the SERT [61]. Interestingly, ERα loses sensitivity with aging in the same manner than SERT [44,45,51,53]. Furthermore, ERα-IGF-R1 sensitivity decreased with aging if an intervention with estradiol did not occur close to OVX [62]. Consequently, it is feasible to consider that both age and time after OVX contribute to explain the lack of sensitivity of females after three weeks of OVX. Taken together it is possible that a complex mechanism of action, which requires further examination, underlies the antidepressant-like action of EE_2_ alone and in combination with CIT.

EE_2_ increased the weight of uteri even though it lacked an effect on the FST. The present data suggest that the beneficial effect of estrogens on the brain is restricted to a specific period close to the natural decline of estrogen levels and this is independent of the peripheral effect of estrogens on different tissues. A shortcoming of the present data is the absence of histological analyses to evaluate the structural changes induced by EE_2_ in the uterus. Furthermore, the differences in the weight of uteri between control groups could be explained by the stimulatory effect of the remaining hormones after OVX. Thus, one week post-OVX, the control uteri were heavier than those obtained after three weeks and in the former EE_2_ did not modify their weight at any dose tested. In contrast, after three weeks, acute EE_2_ administration increased their weight to control values, suggesting that EE_2_ has a physiological action. Specific experiments to analyze a putative stimulatory effect of EE_2_ on uterus weight are warranted.

## 4. Material and Methods

### 4.1. Animals

Female Wistar rats of 15 months were group-housed (five to six per cage) in polycarbonate cages. All animals had free access to food and water. They were maintained on a 12:12-h light:dark cycle with lights on at 10:00 h and a room temperature of 23 ± 2°C. All procedures observed the Mexican Official Norm for animal care and handling (NOM-062-ZOO-1999) and were approved by the Local Institutional Ethics Committee.

### 4.2. Ovariectomy

In the present study all rats were ovariectomized (OVX) one or three weeks before behavioral assessments in order to simulate menopause [63,64,65]. A ventral incision was made to remove the ovaries in rats anesthetized with tribromoethanol (2%; dose: 0.1 mL/kg. i.p.). Care was taken to totally excise the ovaries, which was corroborated by visual inspection. A recovery period of one or three weeks was allowed before rats were randomly assigned to the experimental groups [32].

### 4.3. Drugs

Citalopram clorhidrate (kindly donated by Psicofarma^®^, México, México) was dissolved in physiological saline solution to prepared doses of 1.25, 2.5, 5.0 or 10 mg/kg that were administered in a sub-acute schedule (3 injections/ −23, −5 and −1 h before FST) in a volume of 2 mL/kg. 17 α-ethynyl estradiol (Sigma-Aldrich, Toluca, México) was dissolved in corn oil and administered in a volume of 0.2 mL/rat 48 h before the FST at doses of 1.25, 2.5 or 5 µg/rat. The drugs were freshly prepared. The doses and latencies were chosen from previous studies [40,41,42,43,44,45,46,47,48,49,50,51,52,53,54,55,56,57,58,59,60,61,62,63].

### 4.4. Forced Swim Test

The FST was conducted by placing rats inside individual Plexiglasscylinders (height: 46 cm and diameter: 20 cm) filled with 30 cm of water at 23 ± 2 °C [66,67,68]. Two swim sessions were conducted: a pretest of 15-min followed 24 h later by a 5-min test, which was videotaped for scoring by an observer unaware of the treatments. After each swim session, rats were dried with a towel and placed in heated cages for 30 min before returning them to their home cages.

Three behavioral variables were scored during the test: (1) immobility, defined as the minimal movements done by the animal to keep its head above the water; (2) swimming, identified as gentle movements executed by the rat around the cylinder; and (3) climbing, characterized by vigorous movements of the forepaws directed against the wall of the cylinder [40,67,68].

### 4.5. Experimental Design

#### 4.5.1. Experiment 1: Effect of Post-OVX Interval on the Effect of EE2 or CIT in Middle-Aged Rats on the FST

A dose-response curve for each compound was performed to determine the effective dose of EE_2_ or CIT to decrease immobility in middle-aged OVX rats. Independent groups were tested one or three weeks after the surgery; for CIT the groups were: saline, 1.25, 2.5, 5.0 and 10 mg/kg (*n* = 6–8 per group) and for EE_2_ the groups included were: oil, 1.25, 2.5 and 5.0 µg/rat (*n* = 7–9 per group). CIT was administered −23, −5 and −1 h before the FST. EE_2_ was dissolved in corn oil, prepared 72 h before its administration and injected acutely −48 h before the behavioral test. Doses and latencies were taken from previous data [29,36,65]. At the end of the experiment six rats of each group that received EE_2_ were sacrificed and their uterus was removed, cleaned of fat and weighted in order to obtain an index of the peripheral effect of the steroid [32,37].

#### 4.5.2. Experiment 2: Effect of Post-OVX Interval on the Effect of the Combination of Non-Effective Doses of EE2 Plus CIT in Middle-Aged Rats on the FST

In order to evaluate the effect of the post-OVX interval on the combination of EE_2_ plus CIT, independent groups of OVX rats received: oil/saline, EE_2_ (1.25 µg/rat, −48 h/saline), CIT (2.5 mg/kg; −23, −5, −1 h/oil) and EE_2_ (1.25 µg/rat; −48 h) plus CIT (2.5 mg/kg; −23, −5, −1 h). All groups were tested one (*n* = 6–8 per group) or three weeks (*n* = 6–8 per group) after OVX. The dose of EE_2_ was established in experiment one. Due to the fact that all CIT doses assayed were ineffective in experiment one, the dose chosen for this experiment was 2.5 µg/rat because other SSRIs are effective at this dose when combined with estrogens [21,22,23,24,25,26,27,28,29].

### 4.6. Statistical Analysis

The data are presented as mean ± S.E.M. The data were analyzed using two-way analysis of variance considering time after OVX and treatment as factors. Holm-Sidack tests were used as a post-hoc method of paired comparison. In all cases a *p* < 0.05 was considered significant.

## 5. Conclusions

In conclusion, the present data are in agreement with clinical reports showing that hormonal replacement therapy improves the effects of selective serotonin reuptake inhibitors in the post-menopausal condition [69,70].Importantly, the time of EE_2_ restitution in relation to menopause should be considered in order to reach maximal beneficial effects and reduce the risk of adverse events.

## Figures and Tables

**Table 1:** Effect of ethynyl-estradiol (EE_2_) or citalopram (CIT) on the number (#) of counts of swimming and climbing behavior scored in the forced swim test after one or three weeks post-OVX.

Treatment	Post-OVX Interval	# of Counts of Swimming	# of Counts of Climbing
Oil	One Week	9.3 ± 1.4	9.2 ± 1.0
EE2 1.25 (µg/rat)		13.5 ± 1.6	9.1 ± 2.0
EE2 2.5 (µg/rat)		19.0 ± 3.9	12.5 ± 1.8
EE2 5.0 (µg/rat)		27.1 ± 4.4 **^,##^	7.0 ± 2.1
SAL ^†^		9.3 ± 1.4	10.8 ± 2.6
CIT 1.25		10.3 ± 1.5	7.0 ± 1.6
CIT 2.5		10.7 ± 3.3	6.4 ± 0.7
CIT 5.0		12.0 ± 3.0	8.1 ± 1.2
CIT 10		17.0 ± 3.5	6.8 ± 1.4
OIL	Three Weeks	11.3 ± 2.4	10.0 ± 1.6
EE2 1.25		11.3 ± 2.1	8.1 ± 1.2
EE 2.5		10.3 ± 2.0	7.8 ± 1.6
EE2 5.0		9.1 ± 1.8	10.8 ± 2.0
SAL		10.5 ± 1.8	9.7 ± 2.4
CIT 1.25		7.8 ± 1.8	9.7 ± 2.4
CIT 2.5		9.3 ± 2.3	5.6 ± 1.2
CIT 5.0		16.5 ± 3.9	5.8 ± 2.1
CIT 10		12.2 ± 2.0	11.4 ± 3.2

The data are shown as mean ± S.E.M of the number of counts for swimming and climbing behaviors scored in 5-sec intervals in a 5-min session of forced swimming. ^†^ SAL = saline. Holm-Sidack test; ** *p* < 0.001 *versus* the control group; ^##^ *p* < 0.001 *versus* three weeks.

**Table 2:** Effect of ethynyl-estradiol administration one or three weeks post-OVX on uterus weight (mg).

Ethynyl-Estradiol (µg/rat)	One Week (*n* = 4)	Three Weeks (*n* = 4)
OIL	54.5 ± 8.52	34.5 ± 4.97 ^&^
1.25	53.3 ± 10.5	50.6 ± 1.45
2.5	40.5 ± 3.42	57.2 ± 2.13 *^,&^
5.0	36.0 ± 5.52	62.2 ± 3.11 *^,&&^

The data are presented as mean ± S.E.M of the uterus weight of four rats per group. Holm-Sidack test * *p* = 0.05 *versus* the control group; ^&^ *p* = 0.05, ^&&^ *p* = 0.005 *versus* one week.

**Table 3:** Effect of the combination of ethynyl-estradiol plus citalopram on the number of counts of swimming and climbing behaviors scored in the forced swim test after one or three weeks post-OVX.

Treatment	Time Post-OVX	Swimming	Climbing
OIL/SAL	One Week	8.3 ± 0.68	10.1 ± 1.5
EE2 (1.25 µg/rat)		13.5 ± 1.6	9.1 ± 2.0
CIT (2.5 mg/kg)		10.7 ± 3.3	6.4 ± 0.7
EE2/CIT		24.8 ± 2.5 **^,##^	9.0 ± 2.6
OIL/SAL	Three Weeks	14.2 ± 2.5	8.0 ± 1.8
EE2 (1.25 µg/rat)		11.3 ± 2.1	8.1 ± 1.2
CIT (2.5 mg/kg)		9.3 ± 2.3	9.7 ± 2.4
EE2/CIT		9.8 ± 2.6	7.1 ± 1.4

The data are presented as mean ± S.E.M of the number of counts for swimming and climbing scored in 5-sec intervals in a 5-min session of forced swimming. Holm-Sidack test ** *p* < 0.001 *versus* the control group; ^##^ *p* < 0.001 *versus* three weeks.
